# Long-term outcomes of breast cancer in women aged 30 years or younger, based on family history, pathology and *BRCA1/BRCA2/TP53* status

**DOI:** 10.1038/sj.bjc.6605606

**Published:** 2010-03-16

**Authors:** D G R Evans, A Moran, R Hartley, J Dawson, B Bulman, F Knox, A Howell, F Lalloo

**Affiliations:** 1 Manchester Academic Health Science Centre, Genetic Medicine, St Mary's Hospital, Central Manchester Hospitals Foundation Trust, Manchester M13 9WL, UK; 2 Genesis Prevention Centre, University Hospital of South Manchester and Wythenshawe Hospital NHS Foundation Trust, Manchester M23 9LT, UK; 3 North Western Cancer Intelligence Service, Christie NHS Foundation Trust, Kinnaird Road, Manchester M20 9BX, UK; 4 Department of Pathology, University Hospital of South Manchester and Wythenshawe Hospital NHS Foundation Trust, Manchester M23 9LT, UK; 5 CRUK Department of Medical Oncology, The Christie NHS Trust, University of Manchester, Manchester M20 4BX, UK

**Keywords:** BRCA1, BRCA2, TP53, familial breast cancer, family history

## Abstract

**Background:**

There are relatively few articles addressing long-term follow-up in women with breast cancer at very young ages.

**Methods:**

We have updated and extended our population-based analysis of breast cancer diagnosed at the age ⩽30 years in North-west England to include an extra 15 patients with mutation testing in *BRCA1*, *BRCA2* and *TP53*, with 115 of 288 consecutive cases being tested. Kaplan–Meier curves were generated to assess overall survival, contralateral breast cancer and other second primaries.

**Results:**

Survival analysis of all 288 patients showed poor overall survival, although this improved from a 15-year survival of only 46% in those diagnosed between 1980 and 1989 to 58% in those diagnosed between 1990 and 1997 (*P*=0.05). Contralateral breast cancer rates were at a steady rate of 0.6 per 1000, although the rates in mutation carriers were ∼2 per 1000. Altogether, 16 *BRCA1*, 9 *BRCA2* and 6 *TP53* mutations have now been found among the 115 cases on whom DNA analysis has been performed. BRCAPRO accurately predicted the number of carriers for *BRCA1* and *BRCA2* and was sensitive and specific at the 10 and 20% threshold, respectively. However, BRCAPRO did not seem to give any weight to DCIS, which accounted for two *BRCA1* carriers and three *TP53* carriers and overpredicted mutations at the high end of the spectrum, with only 6 of 11 (54%) with a >90% probability having identifiable *BRCA1/2* mutations.

**Interpretation:**

Rates of new primaries are predicted to some extent by mutation status. BRCAPRO is useful at determining those patients aged ⩽30 years to be tested.

Mutations in *BRCA1*, *BRCA2* and *TP53* account for a proportion of early-onset and familial breast cancer. These mutations confer a lifetime breast cancer risk of 43–85% (Varley *et al*, 1997; Ford *et al*, 1998). Several studies have investigated the frequency of *BRCA1*/2 and *TP53* mutations in families with breast and/or ovarian cancer or Li–Fraumeni (LFS) or LFS-like (LFL) syndrome (Sidransky *et al*, 1992; Varley *et al*, 1997; Gayther *et al*, 1998; Peto *et al*, 1999; Loman *et al*, 2001). Among breast cancer cases unselected for family history (FH), the prevalence of *BRCA1/2* mutations is dependent on the population studied and on the age at diagnosis of malignancy (Fitzgerald *et al*, 1996; Krainer *et al*, 1997; Peto *et al*, 1999; Loman *et al*, 2001). Although these papers have addressed the incidence of *BRCA1/2* in young women with breast cancer, data are still sparse in women diagnosed at very young ages (⩽30 years) and data are lacking for *TP53*.

Lalloo *et al* (2003, 2006) reported on the frequency and penetrance of *BRCA1/2* and *TP53* mutations in early-onset breast cancer in earlier papers. These papers showed a predominance of *BRCA1/2* mutations in familial aggregations of breast cancer, with penetrance estimates of breast cancer as high as 80–90% by 70 years of age. This paper presents a detailed analysis of survival, contralateral breast cancer and other tumour incidence in index cases aged ⩽30 years, both with and without *BRCA1/2* or *TP53* mutations. It also presents results of including an extended series of *BRCA1/2* and *TP53* index cases diagnosed with breast cancer at ⩽30 years of age.

## Methods

### Patients

The 288 original patients were ascertained from a population-based series of consecutive breast cancers from the North Western Cancer Intelligence Service (NWCIS) diagnosed with breast cancer at or under 30 years of age between 1 January 1980 and 31 December 1997. Diagnoses were confirmed using hospital records and pathology reports. Those patients who were proven to have a histological diagnosis other than breast carcinoma were excluded. Patients were approached through their consultant for permission to be interviewed and, after informed consent, to provide blood for DNA testing. A detailed three-generation pedigree was obtained along with copies of hospital notes, where available. Family histories of malignancy were confirmed through the cancer registry, hospital notes or death certificates.

Patients were classified as familial, LFS, LFL or non-familial groups on the basis of FH at initial diagnosis. Familial patients were defined as those with a FH of breast cancer <65 years of age or ovarian cancer at any age in first- or second-degree relatives at the time of the index cases’ primary breast cancer diagnosis. Both LFS and LFL were defined as in Table 1.

A further series of 84 patients diagnosed with breast cancer at or under 30 years of age who had tested positive for mutations in *BRCA1/2* or *TP53* were included for some analyses of contralateral and other tumour risks to enrich the inherited subtypes. Of these patients, 24 were diagnosed in the northwest after 31 December 1997.

Kaplan–Meier (KM) survival curves from diagnosis of breast cancer were derived from the original series alone, with ductal carcinoma *in situ* (DCIS) included and excluded. A comparison between cases diagnosed before and after 1990 was also undertaken. Kaplan–Meier curves were derived for the occurrence of contralateral breast cancer after the initial diagnosis and for other cancer diagnoses following initial breast cancer diagnosis with the inclusion of mutation carriers from the additional data set. Kaplan–Meier curves for contralateral breast cancer from diagnosis were derived using the enriched data set with a confirmed FH or mutation: comparisons were made between no FH (sporadic): *BRCA1*; *BRCA2*; *TP53*; and FH but no mutation.

### Mutation analysis

All samples were screened for mutations in *BRCA1*, *BRCA2* and *TP53* as described previously (Lalloo *et al*, 2003). Since then, further mutation screening of *BRCA1* and *BRCA2* has been undertaken using direct sequencing and multiple ligation-dependent probe amplification, which is a dosage test to detect large single or multiple exon deletions or duplications. An additional 15 samples have become available for mutation testing from the original ⩽30 series.

### Pathology

Pathology was taken from the original pathology report and NWCIS entry. All available tumours from patients who had donated blood samples for DNA analysis were reviewed by a single pathologist (FK). Oestrogen receptor (ER) (6F11 Novocastra, Bannockburn, IL, USA), PR (PGR312 Novocastra) and HER2 (c-erbB2 CB11 Novocastra) immunohistochemistry was performed in cases in which tissue was available. Both ER and PR were scored using the Allred (Quick) score based on the assessment of both proportion and intensity of staining. Her-2 was scored according to standard protocol (Ellis *et al*, 2004). The ER status was considered negative if the Quick score was 0/8 or if <5% cells were positive by immunohistochemistry. Her-2 was regarded as positive if the tumour showed gene amplification by fluorescent *in situ* hybridisation (FISH) or, in the absence of FISH, if the immunohistochemical score was 3+ (scale of 0–3).

### Model prediction

Carrier probabilities of *BRCA1* and *BRCA2* using BRCAPRO and Myriad were calculated using the CancerGene software package (CaGene version 4.3.2) from the University of Texas Southwestern Medical Center (Houston, TX, USA). Additional information about CancerGene is available at http://www.utsouthwestern.edu. The Manchester scoring system was used for comparison (Evans *et al*, 2004). Carrier status was calculated at last follow-up or death of the affected woman to take into account bilateral disease and newer FH. Assessments were made for a 10 and 20% detection rate for each gene and BRCA1/2 combined.

### Statistical analysis

Assessment of KM curves was undertaken by *χ*^2^ test by comparing each grouping separately with all others for *BRCA1*, *BRCA2*, *TP53*, familial mutation negative and sporadic mutation negative. Ninety-five percent confidence intervals (CIs) were derived for survival and contralateral tumour incidence.

## Results

A total of 276 women were registered on the NWCIS with confirmed early-onset primary breast carcinoma diagnosed between 1 January 1980 and 31 December 1997 in the strict regional boundaries. At original ascertainment between 1993 and December 1997, 116 (42%) women were dead and 160 (58%) were alive. As of December 2008, 144 (52%) women were dead and 132 (48%) were alive. The age of diagnosis and grade of tumour did not significantly differ between the living and dead cases. The mean age at diagnosis was 28 years and 3 months (range 18 years 5 months to 30 years 11 months). Consultant permission to approach the patient was refused for 26 living patients (4 subsequently came forward unprompted). Of the remaining 135 cases, 102 consented to participate, 32 refused and 1 could not be traced. Blood samples were available from a further five deceased patients, and were tested after family consent. Further blood samples were obtained from an additional 8 of 12 women affected at the appropriate age and study period on the NWCIS, but outside the strict regional boundaries. As such, genetic status could be established for 115 women from the NWCIS who developed breast cancer at ⩽30 years of age (15 more than our previous report).

In all, 46 patients (42%) had a significant FH, which was consistent with LFS or LFL in 6 cases. A further five women had a FH of breast cancer, which was not consistent with the original study (breast cancer in third-degree relatives or >65 years of age). The remaining 64 cases had no known FH of breast or ovarian cancer at the time of diagnosis and were classified as non-familial.

### Molecular analysis

Overall, pathogenic mutations in *BRCA1*, *BRCA2* and *TP53* were identified in 31 women: 26 of 53 (49%) familial and 5 of 62 (8%) non-familial cases (Tables 2, 3 and 4). Three patients with *BRCA1/2* mutations were sporadic at the time of diagnosis and one patient had only a paternal grandmother with breast cancer aged 65 years. In all four cases, the history changed over follow-up. Two women developed bilateral disease (cases 9 and 16, Table 5) and in the other two, a FH of ovarian cancer and breast cancer developed after their own diagnosis.

Pathogenic *BRCA1* mutations were identified in 16 women (14%) diagnosed with breast cancer at ⩽30 years of age. *BRCA2* mutations were detected in nine women (8%). Pathogenic *TP53* mutations were found in 5 patients (5%) including 3 of 6 (50%) of the LFS/LFL subgroup. The updated analysis demonstrated an additional *TP53* mutation: 659A>G in a family fulfilling LFS criteria, four further *BRCA1* mutations (2682C>T, del exons 1–17, del exons 5–17, 1953DupG) in four familial breast cancers and 1 *BRCA2* mutation (1058C>A) out of the 15 additional samples obtained since our last report (Lalloo *et al*, 2006). The pathology characteristics based on mutation status are presented in Table 3. Table 4 shows a more detailed breakdown of grade 3 tumours. Only in grade 3 triple-negative breast cancer does the rate of *BRCA1* mutation among sporadic cases increase above 10% (2 of 16). However, the overall detection rate in all triple-negative grade 3 cases including those with a FH was 10 out of 27 (37%).

An additional 30 patients with *BRCA1* mutations diagnosed at ⩽30 years of age after 31 December 1979 and 19 with *BRCA2* mutations with the same criteria were identified from our clinic mutation database. In addition, six *TP53* carriers diagnosed at ⩽30 years of age were found. In all, 13 of the total 84 mutation carriers (46 *BRCA1*, 27 *BRCA2*, 11 *TP53*) have had risk-reducing contralateral mastectomy (RRM) representing 20% of the total. We are not aware of any RRMs being performed in the remaining patients.

### Survival

**Figure 1:**
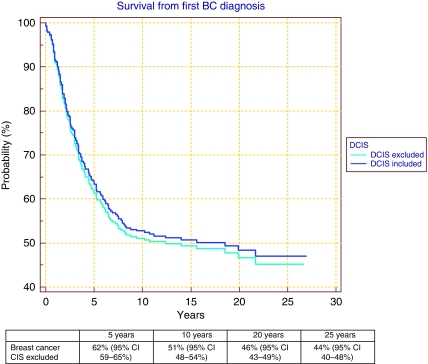
Survival from breast cancer diagnosis in 288 breast cancer cases aged ⩽30 years and diagnosed between 1980 and 1997.

Of the 288 cases in the population-based series, 18 had carcinoma *in situ* as their initial diagnosis. Of these cases, two had comedo DCIS and were shown to have a *TP53* mutation. They subsequently died from a primary glioma and retroperitoneal sarcoma, respectively. Two patients died from subsequent ipsilateral invasive breast cancer and one from non-cancer-related event. Survival analysis from diagnosis in the whole data set is presented in Figure 1. Survival decreases below 50% at the 15-year point. However, this masks a difference between those cases diagnosed before and after 1990. Rates of 5-, 10- and 15-year survival were 69% (95% CI: 65–73%), 58% (95% CI: 54–62%) and 57% (95% CI: 53–61%), respectively, in 133 women diagnosed after 1990 compared with 61% (95% CI: 57–65%), 49% (95% CI: 45–53%) and 46% (95% CI: 42–50%), respectively, in 155 women diagnosed before 1990 (hazard ratio 1.38 *P*=0.05).

### Contralateral breast cancer incidence

**Figure 2:**
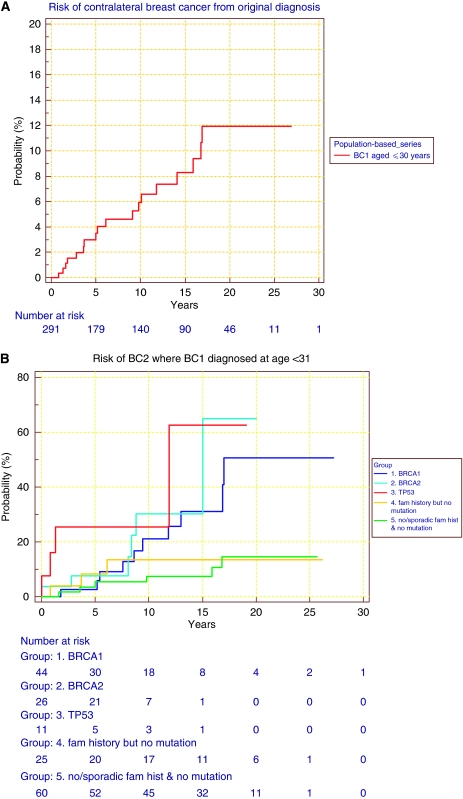
(**A**) Contralateral breast cancer cumulative incidence in all incident breast cancer cases aged ⩽30 years. (**B**) Contralateral breast cancer cumulative incidence in BRCA1, BRCA2 and TP53 breast cancer cases at ⩽30 years of age and in mutation-negative groups.

Contralateral breast cancer incidence is shown in Figure 2A and B. The incidence of contralateral breast cancer up to 20 years of follow-up is 0.6% per year. (Figure 2A). In all, 19 contralateral breast cancers occurred in follow-up (Table 5): 1 in a *TP53* carrier, 4 in *BRCA1* carriers and 1 in *BRCA2*. In the enriched mutation carrier data set, there is a 2% annual risk of contralateral breast cancer up until 15 years, after which the number of patients is too small for any stable estimate (Figure 2B).

### Other cancer incidence

The incidence of non-breast primary cancer was 4.2% at 25 years (Supplementary Table). Surprisingly, no ovarian cancer has so far occurred even among *BRCA1/2* carriers, given the fact that the age of these women now without oophorectomy is 38–55 years. Indeed, in 620.5 years of follow-up in the enhanced data set of 72 women with *BRCA1/2* mutations (censored at date of oophorectomy in 13 women), no ovarian cancers have occurred. Of 59 women with intact ovaries, 25 are now >40 years of age.

### Model performances

Performance of the BRCAPRO model and myriad tables can be seen in Tables 5 and 6. BRCAPRO performed well, especially in unilateral cases, although it seemed to overestimate in bilateral cases (*P*=0.05). In isolated sporadic unilateral cases, the only patient exceeding the 10% threshold (25.8%) with BRCAPRO was a Jewish woman who did not have a mutation. In 11 families with a predicted *BRCA1/2* mutation status >90% (10.47 predicted), only 6 *BRCA1/2* mutations were found (1 in *BRCA2*). Two of the *TP53* carriers had a predicted *BRCA1/2* score of >90% (95.3, 94.3%). The prediction of DCIS cases is also presented in Table 5. Although both the *BRCA1* carriers with DCIS reached the 10% combined threshold, it is clear that this was because of the very strong FH. Indeed, there seems to be no weighting for DCIS, as sporadic cases had a 0% rating for *BRCA1* and even those with a FH were no more likely in BRCAPRO to be a *BRCA1* carrier than an unaffected sibling.

The myriad model did not perform well with poor sensitivity, especially at the 20% level. The Manchester score was partly developed using initial data from this series before the extended follow-up and addition of 15 women. Using the updated pathology-adjusted Manchester score (Evans *et al*, 2009), sensitivity at the 20% level improved from 80 to 88% (Table 6).

## Discussion

This study has updated the previous comprehensive analysis of FH and mutation analysis for all three high-risk breast cancer genes in women diagnosed with breast cancer aged ⩽30 years to include an additional 15 patients with 5 new pathogenic mutations. The analysis of contralateral disease, non-breast primaries and survival has been enriched with additional patients diagnosed at the age of ⩽30 years from our mutation database. We have previously published the prevalence of both *BRCA1/2* and *TP53* mutations in this cohort (Lalloo *et al*, 2003), demonstrating a higher prevalence of *TP53* mutations than previously expected. The mutation detection rate in familial breast cancer in these three genes is ∼50% (26 of 53), demonstrating the importance of accurately documenting a FH when estimating the likelihood of a mutation carrier. Few mutations were found in those women without a FH. These data would therefore not support the testing of *BRCA1*/2 in patients below the age of 31 years without a FH of breast cancer. In particular, even in sporadic cases with grade 3 cancers, the detection rate was only 5%. Among grade 3 triple-negative sporadic cases, the rate of mutation detection was only 12.5% (2 of 16). Lakhani *et al* (2005) reported data on the likelihood of breast cancer being caused by a *BRCA1* mutation by pathological grade and ER status at various ages in women not selected by FH. These data were also from an unselected series. Women aged between 20 and 29 years with grade 3 ER-negative breast cancers had a 35% chance of a *BRCA1* mutation, with a similarly high risk for women aged 30–34 years at 26.5%. Only after the age of 34 years did the risks fall below 10% (Lakhani *et al*, 2005). However, these figures include all women, even those with a FH. Unfortunately, it seems that some clinicians have misinterpreted these data and called for all women <35 years of age with pathological grade 3 and ER-negative breast cancer to be tested, or has offered testing on the basis of a 10–20% testing threshold. Our population-based data confirm a similar 29% risk for young women (age <31 years). This suggests that only triple-negative, pathological grade 3 breast cancers in women <31 years of age qualify for testing, irrespective of FH, on the basis of guidelines using a 10% threshold in outbred populations. Clearly, the CIs are wide on these numbers, and further studies to assess the rates of mutations in sporadic triple-negative patients will help inform models and scoring systems further. For instance, a recent North American study demonstrated mutations in 9% of triple-negative breast cancer patients aged <40 years without, or with minimal, FH of breast or ovarian cancer (Young *et al*, 2009). However, this study did not describe the FH. Nevertheless, use of pathology in our pathology-adjusted Manchester score (Evans *et al*, 2009) improved sensitivity at the 20% threshold by detecting two further mutations at the cost of testing only three further samples.

The data in the current analysis demonstrate the improvement in survival in breast cancer, which is seen in many countries. In the United Kingdom, a more formal approach for breast surgery including axillary node dissection and treatment of all ipsilateral breast tissue (radiotherapy in those undergoing local excision) was highlighted in 1990 (Fentiman and Mansel, 1991). This more formalised treatment schedule may in part explain the significant improvement in survival from diagnosis with time. Nevertheless, overall survival is still poor in this group of young women. This is likely to be related to a more aggressive phenotype with less hormonally sensitive cancers (Figueiredo *et al*, 2007; Anders *et al*, 2008). The poor prognosis seems to particularly apply to breast cancer patients at age <35 years and is consistent between studies in the United Kingdom, North America and Asia (Nixon *et al*, 1994; Jmor *et al*, 2002; Han and Kang, 2010).

The diagnosis of a second primary tumour should prompt the clinician to consider a *TP53* mutation. One patient with a renal carcinoma and a *de novo TP53* mutation went on to develop a sarcoma behind the remaining kidney that was subjected to regular screening radiation from intravenous urograms. An oesophageal cancer also occurred in a *BRCA2* carrier. However, surprisingly, no ovarian cancers occurred in *BRCA1/2* carriers, even though 21 women have lived beyond 40 years of age without an oophorectomy. Population studies have indicated that the subsequent ovarian cancer risk is higher if an individual is diagnosed with breast cancer at a young age, particularly if there is a FH of ovarian cancer (Bergfeldt *et al*, 2002). This is most likely to suggest a high probability of a *BRCA1/2* mutation rather than another mechanism increasing ovarian cancer risk. The data from our study do not suggest a higher ovarian cancer risk in women with known mutations with early-onset breast cancer, compared with mutation carriers with later-onset disease.

This study assessed the performance of mutation prediction models in this population-based data set. We chose to assess the models at last follow-up to determine how the models dealt with new contralateral disease. Direct comparisons with the Manchester scoring system are inappropriate, as this system was partly developed from the original data set at the time of diagnosis. Another model, BOADICEA, also used this data set in development (Antoniou *et al*, 2008). BRCAPRO (Parmigiani *et al*, 1998) performs well, especially in unilateral cases. As no non-Jewish sporadic case (even the adopted individuals) reached the 10% combined threshold even at the time of diagnosis, this supports the hypothesis that, in Western populations, the rates of *BRCA1/2* mutations even in early-onset breast cancer cases without a FH are low. An adopted sporadic case would need to be diagnosed at the age of ⩽24 years to breach the threshold using BRCAPRO. Nevertheless, BRCAPRO does seem to overestimate the *BRCA1/2* probabilities in bilateral cases. It is also interesting to note the handling of DCIS. Although both *BRCA1* carriers with DCIS were identified using the 10% combined threshold, they were as likely to be a carrier as an unaffected sibling. It seems that no weight is given to DCIS to increase either the *BRCA1* or *BRCA2* probability of either the individual or the family in BRCAPRO. Ductal carcinoma *in situ* at the age of ⩽30 years is clearly an important diagnosis, as 45% of the 11 cases had a pathogenic mutation, with DCIS being a particular marker for a *TP53* mutation. The myriad tables (Frank *et al*, 2002) do not have a specific readout for bilateral disease and therefore should probably not be used to assess families with this disease. No patient using the Myriad tables had a score above 39.2% for likelihood of a mutation. There also seems to be a degree of overestimation at the top end of the BRCAPRO model. The model does not allow for sensitivity of mutation techniques, as scores of up to 100% are possible. The model also does not allow for the possibility of other high-penetrance genes, as two of the *TP53* carriers had scores above 94%, yet their FHs were consistent with LFS. We have estimated that a combined score of 40+ using the Manchester score suggests a very high probability of a *BRCA1/2* mutation (Evans *et al*, 2009). Using this threshold, six of seven samples had a detectable mutation.

The strengths of this study are that it is population based and has obtained DNA samples on the great majority of living cases. Overall death rates and second primary rates are secure because of the cancer intelligence service.

This study does have some limitations. The pathology reports on patients diagnosed before 1990 had little information with regard to grade and do not have information regarding hormone receptor status. It was not possible to determine reliable survival data on the basis of tumour grade as a result of this deficiency. However, in the group that submitted a blood sample, a detailed pathological review was possible in most cases.

### Summary

This analysis has shown an improvement in survival for women with very early-onset breast cancer in more recent years. There are high rates of *BRCA1*, *BRCA2* and *TP53* mutations in women aged ⩽30 years with a FH. Among sporadic patients, mutations are generally in those with grade 3 triple-negative tumours. Rates of further primary tumours other than contralateral breast cancer are not high, except in *TP53* carriers, and very young-onset *BRCA1/2* carriers do not seem to be at enhanced risk of ovarian cancer compared with other *BRCA1/2* carriers. Contralateral breast cancer rates seem stable at ∼0.6% annually in all women and at 2–3% in mutation carriers.

## Figures and Tables

**Table 1:** Diagnostic criteria for Li–Fraumeni syndrome and Li–Fraumeni-like syndrome

**Li–Fraumeni syndrome (Li *et al*, 1988)**	**Li–Fraumeni-like syndrome (Birch *et al*, 1994)**
Proband <45 years with a sarcoma	Proband <45 years with childhood tumour, sarcoma, brain tumour or adrenocortical tumour
Plus first-degree relative <45 years with any cancer	Plus first- or second-degree relative in the same lineage with typical LFS tumour at any age
Plus additional first- or second-degree relative in the same lineage aged <45 years with any cancer or a sarcoma at any age	Plus another first- or second-degree relative in the same lineage with any cancer <60 years

Abbreviation: LFS=Li–Fraumeni syndrome.

**Table 2:** Histology and vital status on 276 incident breast cancer patients ⩽30 years and 12 additional cases from the NWCIS

	**Total**	**Alive (%)**	**Deaths from breast or other cancer**
IDC grade 3 ER negative	40	30 (75%)	10
IDC grade 3 ER positive	19	15 (79%)	4
IDC grade 3 no receptor status	60	25 (42%)	35
All grade 3	**119**	**70 (59%)**	**49**
IDC grade 2	37	14 (38%)	23
IDC grade 1	9	4 (45%)	5
DCIS	18	13 (72%)	5
IDC no grade on pathology report	90	35 (39%)	53
Lobular invasive	8	4 (50%)	4
Scirrhous	3	1 (33%)	2
LCIS	2	2 (100%)	0
Spheroidal	2	0 (0%)	2
Mucinous	1	0 (0%)	1
SCC nipple	1	1 (100%)	0
Total	**288**	**144**	**144**

Abbreviations: DCIS=ductal carcinoma *in situ*; ER=oestrogen receptor; IDC=invasive Ductal Carcinoma; LCIS=Lobular Carcinoma *in situ*; NWCIS=North Western Cancer Intelligence Service; SCC=Squamous Cell Carcinoma.

**Table 3:** Pathology characteristics of 115 patients with DNA testing

	**Total**	***BRCA1***	***BRCA2***	***TP53***	**Any mutation**
IDC grade 3 ER negative	35	10	0	0	10/35
IDC grade 3 ER positive	16	1	4	0	5/16
IDC grade 3 no receptor status	16	2	2	0	4/16
IDC grade 2	15	0	2	0	2/15
IDC grade 1	4	0	0	0	0/4
DCIS	11	2	0	3	5/11
IDC no grade	10	0	0	1	1/10
Lobular invasive	4	1	0	0	1/4
LCIS	2	0	0	0	0/2
Spheroidal	2	0	1	1	2/2
Mucinous	1	0	0	0	0/1
SCC nipple	1	0	0	0	0/1
Total	115	16 (14%)	9 (8%)	5 (5%)	29/115 (25%)

Abbreviations: DCIS=ductal carcinoma *in situ*; ER=oestrogen receptor; IDC=invasive Ductal Carcinoma; LCIS=Lobular Carcinoma *in situ*; SCC=Squamous Cell Carcinoma.

**Table 4:** Frequency of constitutional *BRCA1* and *BRCA2* mutation in grade 3 IDC with oestrogen receptor status and triple negative status in a population based study of breast cancer patients ⩽30 years

	**All IDC grade 3 ER negative**	**All IDC grade 3 triple negative**	**Grade 3 IDC no hormone receptor status available**	**Grade 3 ER positive**	**All Grade 3**	**All breast cancer cases**
*BRCA1: all cases*	10/35 (29%)	10/27 (37%)	2/16 (14%)	1/16 (6%)	13/67 (19.5%)	16/115 (14%)
Sporadic	2/22 (9%)	2/16 (12.5%)	0/7	0/11	2/40 (5%)	2/62 (3%)
Familial	8/13 (61%)	8/11 (73%)	2/9 (22%)	1/5 (20%)	11/27 (41%)	14/53 (26%)
*BRCA2: all cases*	0/35	0/27	2/16 (12%)	4/16 (25%)	6/67 (9%)	9/115 (8%)
Sporadic	0/22	0/16	1/7 (14%)	0/11	1/40 (2.5%)	1/62 (1.5%)
Familial	0/13	0/11	1/9 (11%)	4/5 (80%)	5/27 (18%)	8/53 (15%)

Abbreviations: ER=oestrogen receptor; FISH=fluorescent *in situ* hybridisation.

In all, 37 of 67 (55%) grade 3 tumours had HER2 status assessed on pathology review. Eight (22%) were HER2 positive on FISH analysis.

**Table 5:** Performance of BRCAPRO and Myriad models in predicting BRCA1/2 mutation status

	**Number**	**BRCAPRO *BRCA1***	**BRCAPRO *BRCA2***	**BRCAPRO combined**	**Myriad**	***BRCA1* actual**	***BRCA2* actual**	**Combined *BRCA1*+*BRCA2***
Sporadic breast cancer	64	3.47	2.14	7.6	4.2	2	1	3
Sporadic breast cancer unilateral	54	1.1	1	2.1	3.6	1	1	2
Bilateral breast cancer	17	7.6	3.0	10.6	2.2	4	1	5
Bilateral sporadic	8	2.35	1.15	3.5	0.5	1	0	1
Bilateral family history positive	9	5.3	1.8	7.1	1.7	3	1	4
Familial unilateral	43	10.4	4.4	14.8	6.4	11	7	18
DCIS	11	0.60	0.35	0.95	0.4	2	0	2
Total	**115**	**19.2**	**8.4**	**27.6**	**12.4**	**16**	9	25

Abbreviation: DCIS=ductal carcinoma *in situ*.

**Table 6:** Sensitivity and specificity of BRCAPRO and Myriad tables at the 10 and 20% level for *BRCA1*, *BRCA2* and both genes combined

**Model/scoring system**	**Sensitivity**	**Specificity**	**Positive predictive value**	**Negative predictive value**
BRCAPRO combined 10%	23/25 (92%)	65/90 (72%)	23/48 (48%)	65/67 (97%)
BRCAPRO combined 20%	20/25 (80%)	73/90 (81%)	20/37 (54%)	73/78 (94%)
BRCAPRO BRCA1 10%	14/16 (87.5%)	72/99 (72%)	14/41 (34%)	72/74 (97%)
BRCAPRO BRCA2 10%	4/9 (44%)	78/106 (74%)	4/32 (12.5%)	78/83 (94%)
Myriad 10%	18/25 (72%)	76/90 (84%)	18/32 (56%)	76/83 (92%)
Myriad 20%	8/25 (32%)	86/90 (96%)	8/12 (75%)	86/103 (83%)
MANCHESTER combined 10%	25/25 (100%)	57/90 (63%)	25/58 (43%)	57/57 (100%)
MANCHESTER combined 20%	20/25 (80%)	77/90 (86%)	20/33 (60%)	77/82 (94%)
MANCHESTER BRCA1 10%	15/16 (94%)	73/99 (73%)	15/41 (37%)	73/74 (99%)
MANCHESTER BRCA2 10%	6/9 (67%)	78/106 (74%)	6/34 (18%)	78/81 (96%)
