# Supplementary material for: Long-term outcomes of breast cancer in women aged 30 years or younger, based on family history, pathology and BRCA1/BRCA2/TP53 status
Source: Br J Cancer. 2010 Mar 16;102(7):1091–8. doi: 10.1038/sj.bjc.6605606 (PMC2853095; doi:10.1038/sj.bjc.6605606)
Supplement: Supplementary Table [file 6605606x1.doc]

Supplementary Table: Contralateral and other new primary cancers

| Case | age at first primary | breast cancer 1 | status | family history | contralateral | age contra-lateral | mutation | age other primary | Other primary type |
| --- | --- | --- | --- | --- | --- | --- | --- | --- | --- |
| 1 | 29 | IDC grade 3 | dead 37 | No | Comedo DCIS | 30 | TP53 | 31/36 | Renal RCC/ sarcoma retroperitoneal |
| 2 | 30 | IDC | dead 33 | Unknown | DCIS | 31 | unknown |  |  |
| 3 | 30 | LCIS | alive 45 | Unknown | IDC grade 3 | 39 | unknown |  |  |
| 4 | 30 | IDC grade 3 | alive 49 | Unknown | IDC | 44 | unknown |  |  |
| 5 | 28 | IDC | dead 46 | FHBr | Grade 3 medullary | 31 | Negative |  |  |
| 6 | 30 | IDC grade 2 | dead 36 | FHBr | IDC grade 2 | 33 | BRCA2 |  |  |
| 7 | 29 | grade 3 medullary | alive 47 | Unknown | IDC grade 3 | 39 | unknown |  |  |
| 8 | 29 | IDC grade 3 | alive 47 | FHBr | IDC grade 3 | 34 | BRCA1 |  |  |
| 9 | 29 | Lobular | alive 46 | FHBr | IDC grade 3 | 37 | BRCA1 |  |  |
| 10 | 30 | IDC grade 3 | alive 50 | No | IDC grade 2 | 46 | Negative |  |  |
| 11 | 22 | IDC grade 2 | alive 34 | FHBr | IDC grade 3 | 28 | Negative |  |  |
| 12 | 29 | IDC grade 2 | dead 36 | No | IDC grade 3 | 32 | Negative |  |  |
| 13 | 28 | IDC | alive 49 | FhBrOv | Grade 3 atypical medullary | 45 | BRCA1 |  |  |
| 14 | 27 | lobular | alive 45 | No | IDC grade 2 | 42 | Negative |  |  |
| 15 | 25 | IDC grade 3 | alive 42 | Unknown | IDC grade 3 | 35 | unknown |  |  |
| 16 | 30 | IDC grade 3 | alive 53 | No | Grade 3 medullary | 42 | BRCA1 |  |  |
| 17 | 28 | IDC | alive 41 | No | IDC grade 2 | 38 | Negative |  |  |
| 18 | 30 | IDC grade 2 | alive 43 | No | Grade 3 lobular | 36 | Negative |  |  |
| 19 | 26 | IDC grade 3 | dead 28 | No | IDC grade 3 | 27 | Negative |  |  |
| 20 | 30 | IDC grade 3 | dead 50 | No | n/a | n/a | Negative | 50 | Rectum |
| 21 | 28 | IDC | alive 50 | FH Br/ov | n/a | n/a | BRCA2 | 39 | Oesophagus |
| 22 | 28 | IDC | dead 39 | unknown | n/a | n/a | unknown | 39 | AML |
| 23 | 30 | IDC grade 2 | Alive 43 | unknown | n/a | n/a | unknown | 39 | Parotid mucoepidermoid carcinoma |
| 24 | 29 | IDC grade 2 | Alive 44 | No | n/a | n/a | Negative | 17 | Hodgkins |
